# Markers in Acute Coronary Syndrome: Distal Coronary Embolism at Percutaneous Coronary Intervention

**DOI:** 10.3390/jcdd12080315

**Published:** 2025-08-19

**Authors:** Niya Emilova Semerdzhieva, Simeon Dimitrov, Adelina Tsakova, Mariana Gospodinova, Petar Atanasov, Vesela Lozanova

**Affiliations:** 1 Clinic of Internal Medicine, University Emergency Hospital ‘Pirogov’, 1606 Sofia, Bulgaria; 2 Clinic of Cardiology, Department of Interventional Cardiology, Hospital ‘Hristo Botev’, 3001 Vratza, Bulgaria; simeon.dtr@gmail.com; 3 Clinical Laboratory Department, University Hospital ‘Sofiamed’, 1680 Sofia, Bulgaria; 4 Expert Amyloidosis Centre, University Hospital ‘St. Ivan Rilski’, 1431 Sofia, Bulgaria; 5 Department of Biochemistry, Medical University, 1431 Sofia, Bulgaria; vlozanova@medfac.mu-sofia.bg

**Keywords:** acute coronary syndrome, percutaneous coronary intervention, distal coronary embolism, TIMI flow grade, myocardial blush grade, triglycerides, dehydroepiandrosterone sulfate

## Abstract

(1) Introduction: Distal coronary emboli occur in up to 15–30.5% of patients undergoing percutaneous coronary intervention (PCI) for acute myocardial infarction (AMI) and are associated with poor myocardial reperfusion in the territory of the infarct-related artery. The objective of this study was to analyze the possible laboratory, clinical and imaging indicators of distal coronary embolism detected with an angiography at the time of PCI with stent implantation for acute coronary syndrome (ACS). (2) Methods: This analysis included 137 patients with ACS. The levels of cardiac enzymes (creatine kinase [CK], muscle–brain fraction of CK, high-sensitivity troponin T [hsTnT]), inflammatory markers (high-sensitivity C-reactive protein, white blood cell counts), sex steroids (total 17β-estradiol, total testosterone, dehydroepiandrosterone sulfate [DHEA-S]), serum lipids and oxidized low-density lipoproteins (oxLDL) were measured and analyzed for their relationship with the incidence of distal coronary embolism at PCI. (3) Results: Slow coronary blood flow was detected in the coronary artery subject to intervention in 9.4% (*n* = 13) of patients. Triglyceride (TG), high-density lipoprotein (HDL), glucose and serum DHEA-S levels were found to be associated with distal coronary embolization and slow coronary flow at PCI with stenting (DHEA-S: 1.316, OR 1.044–1.659, *p* = 0.020; TG: 1.130, OR 0.990–1.300, *p* = 0.072; HDL: 2.326, OR 0.918–5.8977, *p* = 0.075; glucose: 1.130, OR 0.990–1.300, *p* = 0.072). In the multivariable model, only DHEA-S after PCI tended to indicate a risk of distal coronary embolism (DHEA-S: *p* = 0.071; TG: *p* = 0.339; glucose: *p* = 0.582; HDL: *p* = 0.502). (4) Conclusions: Patients with ACS with higher triglyceride levels are at risk of developing slow blood flow after percutaneous intervention with stent implantation. Elevated DHEA-S possibly reflects sympathoadrenal and hypothalamus–pituitary–adrenal hyperactivity associated with ACS and coronary intervention.

## 1. Introduction

Distal coronary embolization occurs in up to 15–30.5% of patients undergoing percutaneous coronary intervention (PCI) for ST elevation acute myocardial infarction (MI) and is associated with poor myocardial reperfusion in the territory of the infarct-related artery (IRA; less often postprocedural thrombolysis in myocardial infarction [TIMI] 3 flow and postprocedural myocardial blush grade [MBG] 2–3) [1,2,3,4]. Patients with distal embolization are diagnosed with larger infarct size and an unfavorable prognosis [1,2,3,4]. They are older, with a larger prevalence of diabetes, previous MI and advanced class of heart failure at presentation. Distal coronary embolization is also more often observed in females and in patients with right infarct-related coronary artery, high thrombus burden, longer infarct-related lesion, less frequent direct stenting and less use of bare metal stents [1,5,6]. Crucial factors of slow coronary flow/no-reflow after percutaneous coronary intervention for myocardial infarction include high thrombotic load, larger plaque area and greater remodeling of the infarct-related artery [6,7,8]. Leukocytosis and high neutrophil-to-leukocyte ratio and platelet count at admission are independent predictors of periprocedural microcirculatory injury with angiographically impaired reperfusion after primary percutaneous intervention [9,10]. Hyperglycemia is associated with impaired microvascular function. The prognostic impact of reduced baseline renal function on myocardial reperfusion after primary PCI begins with the slightest decrease in glomerular filtration rate (GFR < 90 mL/min/m^2^) and increases proportionally with a further decrease in glomerular filtration rate [9,10].

Transient slow coronary flow is slow coronary flow during coronary intervention which regains its normal characteristics (angiographic TIMI grade 3 flow) at the completion of the procedure. It is encountered in about 10% of patients with coronary interventions. The transient no-reflow is also associated with increased in-hospital and six-month mortality [11]. The extent of embolization-induced myocardial damage by primary PCI is in many cases too small to be detected by available measurement techniques; thus, it is frequently considered as not clinically relevant [12]. However, the embolic complications generated by PCI of saphenous venous grafts are more frequent [12], and the embolic complications of carotid PCI are associated with serious sequelae [12]. The results of these studies have yielded valuable data for practice.

The objective of this study was to analyze the possible laboratory, clinical and imaging indicators of distal coronary embolism detected at angiography at the time of percutaneous coronary intervention with stent implantation in patients with acute coronary syndrome (ACS).

## 2. Materials and Methods

### 2.1. Study Population

This is a cross-sectional sub-study which included 137 patients (36.5% women) admitted with a diagnosis of ACS at the “Alexandrovska” University Hospital in the period 2011–2014. The data of each patient were recorded in a protocol approved by the hospital ethics committee. This study was retrospectively registered in the UK’s Clinical Study Registry (ISRCTN) with registration number ISRCTN62480360. The spectrum of ACS in this study included acute myocardial infarction with persistent ST elevation (STEMI), acute myocardial infarction without persistent ST elevation (NSTEMI) and unstable angina (UA). UA was defined as myocardial ischemia at rest or on minimal exertion in the absence of acute cardiomyocyte necrosis. It was characterized by prolonged (>20 min) angina at rest; new onset of severe angina; angina that is increasing in frequency, longer in duration or lower in threshold; or angina that occurs after a recent episode of MI. NSTEMI was defined as a condition with symptoms of ongoing myocardial ischemia, ECG abnormalities including ST depression and T wave changes (especially biphasic T waves or prominent negative T waves) and increase in high-sensitivity cardiac troponin T five times over the upper reference limit. All patients diagnosed with STEMI presented with ischemic chest pain lasting more than 30 min in combination with persisting ST elevation > 1 mm in at least two (adjacent) ECG leads or persisting ST depression and T wave inversion in leads pointing to one coronary artery territory and enzyme elevation (CK-MB more than twice the norm or high-sensitivity cardiac troponin T: more than five times the upper reference limit) [13].

Strict exclusion criteria were met: coronary dissections; cases of cardiogenic shock; aortocoronary bypass; infarction of the left main coronary artery; technically poor-quality echographic or angiographic recordings; inability to determine the infarcted artery; simultaneously developing infarction of more than one coronary artery; incomplete revascularization of the infarcted artery—significant remaining stenoses of the infarct-related artery (IRA) after PCI, technically impossible for revascularization. Other exclusion criteria were as follows: acute infectious disease, chronic inflammatory disease (including rheumatologic disease), known or suspected neoplastic processes, surgical procedures and trauma within two weeks before hospital admission, and disease of adrenal gland and pituitary gland.

**Figure 1:**
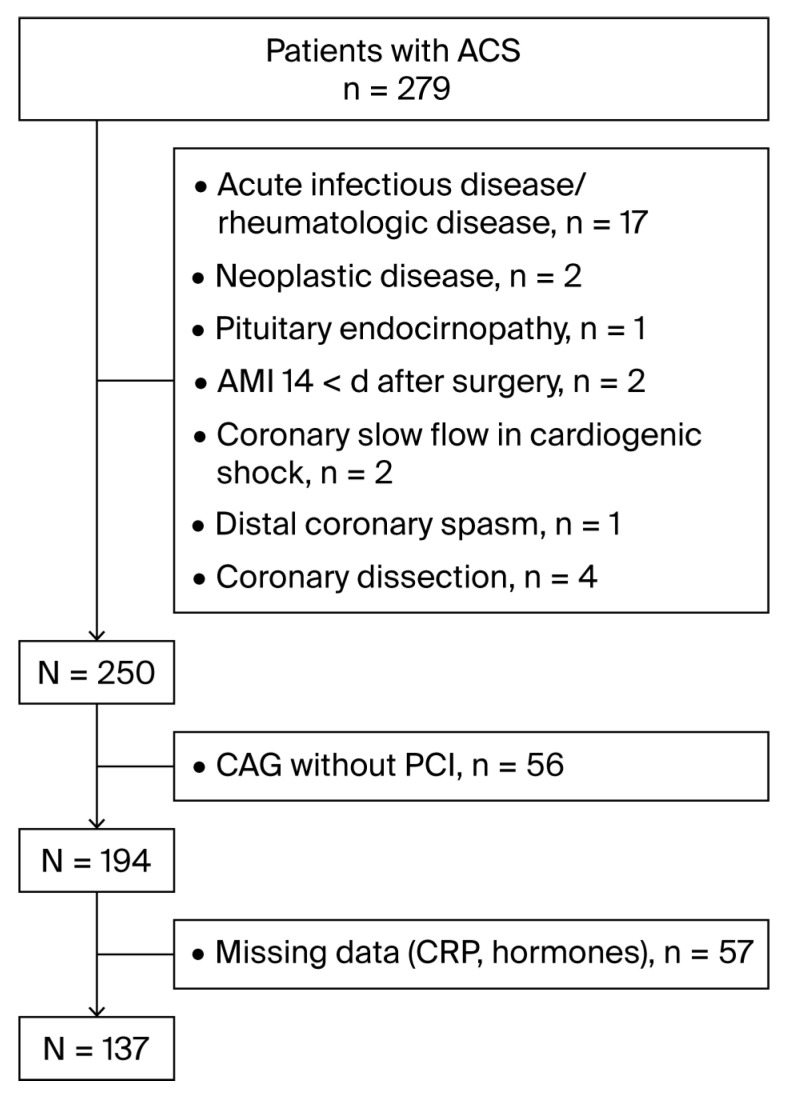
Flowchart of enrollment of the patients. ACS, acute coronary syndrome; AMI, acute myocardial infarction; CAG, coronary arteriography; PCI, percutaneous coronary intervention; DM, diabetes mellitus; CRP, C-reactive protein.

The exclusion criteria and the rejected number of subjects based on each are displayed in Figure 1.

### 2.2. Laboratory Assessment

The levels of cardiac enzymes (creatine kinase [CK], muscle–brain fraction of CK [CPK-MB], and high-sensitivity troponin T [hsTnT]), inflammatory markers (high-sensitivity C-reactive protein [hsCRP], white blood cell [WBC] counts), sex steroids (total 17β-estradiol [E2], total testosterone [T], dehydroepiandrosterone sulfate [DHEA-S]), serum lipids and oxidized low-density lipoproteins [oxLDL] were measured and analyzed for their relationship with the incidence of distal coronary embolism at PCI for ACS. Venous blood samples were drawn within six hours after PCI. The samples were collected into EDTA sample tubes, centrifuged at 12,000 rpm for 20 min, and stored at −20 °C until analysis. The hsCRP concentrations were determined using a latex-enhanced immunoturbidimetric assay (Roche Diagnostics GmbH, Manheim, Germany) on the COBAS INTEGRA 700 analyzer [14]. The levels of the steroid hormones and hsTnT were assessed using an electrochemiluminescent immunoassay with Roche Diagnostics reagents on the Elecsys 2010 analyzer [15]. These methods have been detailed elsewhere. Plasma levels of oxLDL were quantified using the OxiSelect Human Oxidized LDL immunosorbent assay (ELISA; MDA-LDL) kit (Cell Biolabs, San Diego, CA, USA) and a sandwich ELISA [16].

### 2.3. PCI Procedure

The recanalization of the infarcted artery using primary angioplasty was performed up to 12 h after the onset of the infarction. Percutaneous intervention in unstable angina cases was performed within up to 48 h of hospital admission based on symptoms, electrocardiographic (ECG) and laboratory indicators of high risk of adverse events.

Coronary arteriography and percutaneous coronary intervention with stent implantation were performed from femoral access on a Siemens Coroscop device (Siemens, Munich, Germany). A heparin bolus of 60 U/kg was administered intravenously during the procedure. 6F and 7F PCI guiding catheters were used. Coronary angioplasty was performed using the Monorail technique and 0.014˝ × 180–195 cm PCI guidewires—floppy, standard or hydrophilic. Lesions of non-infarcted vessels were not dilated at the same stage; non-infarcted stenoses of the infarcted artery were dilated when significant. A stent was used in all LAD infarctions. Intracoronary glyceryl trinitrate (GNT) was used in one or more bolus doses of 0.125–0.2 mg in all patients with systolic blood pressure above 95 mm Hg. Procedural success was defined as TIMI blood flow after PCI 3 or 2 and residual stenosis < 10% with stent. An acquisition rate of 12.5 frames per second and digital recording with synchronous ECG during contrast injection were used. The results were recorded on CD. Collaterals to the IRA were assessed according to the Rentrop135 scale in an appropriate projection with a sufficiently long acquisition for good imaging. In all patients on the day of PCI, aspirin 250–500 mg was used before the procedure and clopidogrel 300–450 mg (at the discretion of the operators—before or after PCI) with stent placement. After PCI with stent placement, routine terms for clopidogrel and aspirin therapy were recommended. The IIb/IIIa receptor inhibitor eptifibatide was used at the discretion of the operators. Intracoronary boluses of verapamil, eptifibatide, GTN and/or nitroprusside were used in cases of complicated procedure with compromised coronary microcirculation. Coronary disease severity was assessed by calculating the SYNTAX score (https://syntaxscore.org/calculator/syntaxscore/frameset.htm accessed on 4 May 2025) and Gensini score for each patient [17].

Distal coronary embolism during PCI was evaluated with visual assessment of thrombus dislodging at angiography and an impairment in epicardial coronary flow and myocardial perfusion. TIMI coronary grade flow was used for the assessment of consistency of epicardial perfusion at coronary angiography. TIMI grade flow 0 represented total occlusion: no antegrade flow beyond the point of occlusion. Grade 1 designated contrast material which passes beyond the area of obstruction but fails to opacify the entire coronary bed distal to the obstruction for the time of angiographic filming. As grade 2 was defined as coronary flow that enters into or clears from the distal bed slower than its entry into or clearance from comparable areas. Grade 3 epicardial coronary flow was flow as rapid as antegrade flow into the bed proximal to the obstruction [18].

The microvascular perfusion was evaluated with the myocardial blush grade, a method of visual assessment of the intensity of staining myocardial tissue with angiographic contrast (called “myocardial blush”) after the obstruction has been resolved [19]. The MBG is scored from 0 to 3 (Table 1).

### 2.4. Echocardiography

Echocardiography (with harmonic fusion) was performed at admission, using HP-SONOS-5500 or Aloka ProSound 10 (Hitachi Aloka Medical, Hitachinaka, Japan) ultrasound systems. The recordings were stored for potential image restoration and repeated analysis. Regional kinetics was assessed according to the 16-segment model. A four-point scale was used to assess left ventricular kinetic disorders: 1—normo- or hyperkinesia; 2—hypokinesia; 3—akinesia; 4—dyskinesia or aneurysm. Echography was performed with the obligatory use of the parasternal view: the short axis—basal and middle (at the level of the papillary muscles); and two apical views—four- and two-chamber. Where possible, an apical view along the short axis and a longitudinal parasternal view were also recorded, when necessary, to assess left ventricular kinetics.

### 2.5. Statistical Analysis

The distribution of the variables was tested using the Kolmogorov–Smirnov and Shapiro–Wilk tests. The associations between variables were analyzed using parametric (independent samples t-test) and non-parametric (χ2 test, Fisher’s exact tests, and Mann–Whitney U test) methods for patients with normal and abnormal distribution, respectively. All variables were further checked for significant associations with the incidence of distal coronary embolism using Cox proportional regression univariable and multivariate analyses. The predictors of distal coronary embolism explored in the univariable model were age, estradiol, the estradiol-to-testosterone ratio, DHEA-S, white blood cell count, platelet count, body mass index, C-reactive protein, the extent and severity of coronary atherosclerosis (SYNTAX score; Gensini score), glucose, serum lipids, oxLDL, triglyceride–glucose index and left ventricular ejection fraction. The peak values of cardiac enzymes were tested in addition to the abovementioned variables as indicators of the highest endogenous levels of DHEA-S in the univariable model. The multivariable model included the significant predictors of distal coronary embolism and the highest DHEA-S yielded in the univariable analysis. The analyses were conducted using MedCalc statistical software version 23.2.0 (MedCalc Software Ltd., Ostend, Belgium). A two-tailed p-value of less than 0.05 was considered statistically significant.

## 3. Results

Slow coronary flow and distal coronary embolization during PCI were detected in the coronary artery of 9.4% (*n* = 13) of our patients with ACS (Table 2).

The levels of DHEA-S and E2 in healthy or minimally diseased patients, and control subjects (*n* = 26) were not statistically different from patients with ACS, while E2/T was significantly higher in patients with ACS (DHEA-S: 3.1 ± 2.3 vs. 3.8 ± 2.3 µmol/L, *p* = 0.226; E2: 133.4 ± 93.4 vs. 145.8 ± 109.7 pmol/L, *p* = 0.560; E2/T: 0.6 ± 1.2 vs. 0.2 ± 0.3 nmol/L, *p* = 0.002).

**Figure 2:**
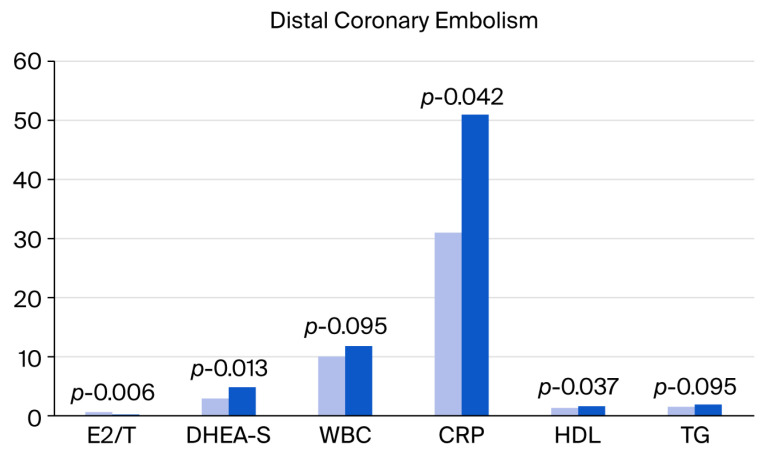
Variables associated with distal coronary embolism at PCI in acute coronary syndrome. The abbreviations are the same as those used in Table 2.

**Figure 3:**
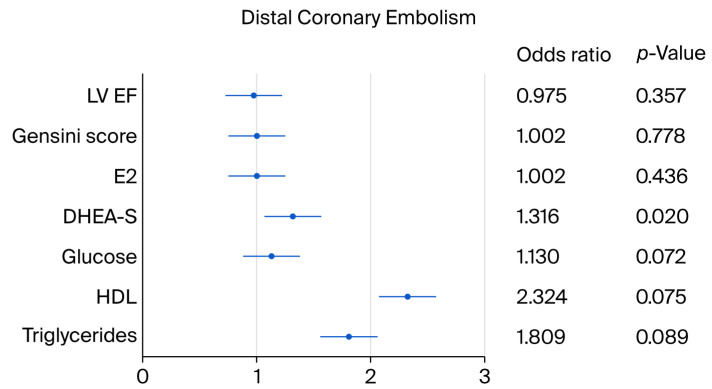
Indicators of distal coronary embolism at PCI in acute coronary syndrome. The abbreviations are the same as those used in Table 1.

In the univariable analysis, the serum DHEA-S levels and a tendency toward triglycerides, HDL and glucose showed a relationship with the incidence of distal coronary embolization and coronary slow flow after PCI in ACS (Table 3, Figure 2 and Figure 3).

DHEA-S was the only statistically significant predictor (*p* = 0.039) according to the multivariable stepwise regression analysis (*p* = 0.039).

The patients with complicated PCI had significantly higher cardiac enzyme values measured up to six hours after PCI compared to the rest, indicating periprocedural myocardial injury (CPK: 755.5 ± 1168.2 vs. 1924.1 ± 1401.7 U/l, *p* = 0.001; OR 1.001, 95% CI 1.0001–1.001, *p* = 0.007; CPK-MB: 76.5 ± 103.5 vs. 105.5 ± 262.2 U/l, *p* = 0.0001; OR 1.006, 95% CI 1.002–1.009, *p* = 0.001; troponin T: 1.9 ± 3.0 vs. 3.4 ± 2.6 ng/mL, *p* = 0.085; OR 1.137, 95% CI 0.975–1.326, *p* = 0.102). In contrast to hsTnT, CK-MB showed significant discriminative ability for assessment of the injury of myocardium associated with a complicated PCI procedure in the setting of ACS (multiple regression model: CK-MB: *p* = 0.001; hsTnT: *p* = 0.346; CK: *p* = 0.787).

Variables indicating higher levels of DHEA-S are presented in Table 4.

Age (*p* = 0.0006), E2/T (*p* = 0.012) and CK-MB (*p* = 0.035) indicated the highest quartiles for DHEA-S in the stepwise multivariable model. Detailed characteristics of the cases of distal coronary embolism was included in Table A1.

## 4. Discussion

Myocardial blush grading (MBG) is a method of visually assessing the intensity of myocardial tissue staining with angiographic contrast (called “myocardial blush”) after a thrombotic coronary obstruction associated with ruptured coronary plaque has been resolved [19]. The TIMI Myocardial Perfusion Grade (TMPG), on the other hand, considers how quickly the contrast agent clears from the heart muscle [20]. Both are used to assess myocardial perfusion [21]. Both improved epicardial flow and myocardial perfusion were independently associated with improved survival after STEMI [20]. Patients with TIMI perfusion grade 3, in particular, showed greater global ejection fractions, smaller enzyme peaks and a trend toward a lower morbidity index compared with patients with grades 0–2 [22]. Conversely, a post-PCI increase in serum cardiac troponin I > 40% combined with an absolute postprocedural value ≥ 5 times the upper reference limit was identified as the threshold for diagnosing myocardial injury associated with PCI in patients with myocardial infarction. Recognizing these events helps chiefly in the improvement of prognosis and management of patients with NSTEMI [23].

In our study, the patients with ACS, distal coronary embolism and transiently reduced blood flow in infarct-related artery after PCI showed higher baseline DHEA-S and a tendency toward higher triglyceride levels, higher HDL cholesterol and higher glucose after PCI.

DHEA-S was significantly inversely associated with age and plasma triglyceride concentration in the general population [24]. Thus, it is not clear why TG, TG-glucose index and CRP were positively related to the levels of DHEA-S and the cases of distal coronary embolism in our study. In both men and women, significantly elevated DHEA and DHEA-S levels were observed in response to the stressor [25]. They were positively associated with the magnitude of the changes in ACTH, cortisol and heart rate [25]. Studies have also demonstrated a positive association between psychological stress, cortisol, inflammation and serum triglyceride levels [26,27]. DHEA-S is a precursor of gonadal steroids outside of reproductive age. DHEA-S along with E2 levels, E2/T and plasma TGs reflect activation of the sympathetic nervous system and hypothalamus/pituitary/adrenal axis in the acute phase of MI. Accordingly, post-PCI myocardial injury (CK-MB) via pro-inflammatory pathways and increased aromatase expression correlated positively with DHEA-S levels. The activation adrenal steroid production at the time of ACS (an acute critical disease) in turn may serve as a mechanistic explanation of the positive relationship between the highest DHEA-S and E2/T [28]. In male patients, any critical illness leads to a decrease in testosterone levels, coupled with increased levels of estradiol. The decline in testosterone in critical illness is primarily due to (1) inflammation impairing androgen synthesis and (2) increased aromatization of T to E2 in peripheral tissues [29]. In females, critical illness leads to an increase in testosterone and estradiol levels. This elevation is attributed to (1) adrenal activation and (2) increased aromatization [29].

Acute hypertriglyceridemia causes endothelial dysfunction via enhanced oxidant stress [30]. The elevated fasting triglyceride levels are associated, although mildly, with increased severity of angiographic CAD in patients undergoing therapy with statins [31]. A higher plaque lipid index > 4 mm (lipid-rich lesions) independently determines the risk of distal embolization and reduced blood flow after PCI [32,33]. Balloon or stent expansion causes the rupture of the lipid core of the infarct-related plaque; the lipid fraction activates thrombus formation. These thrombotic fragments can compromise distal coronary microcirculation. Pre-interventional optical coherence tomography (OCT) studies have suggested a possible association between lipid-rich plaque and the phenomenon of slow blood flow in IRA at PCI, especially for cases of slightly higher than usual balloon dilation pressure during stenting [8]. Patients with high thrombus burden and distal embolization might have lower triglycerides if they are undergoing therapy with statins [5]. A study has shown that intensive TG reduction along with TC and LDL-C lowering with PCSK9 inhibitor gradually provide additional deferred improvement in coronary flow in IRA 6 months after coronary angioplasty for acute coronary syndrome [34].

The interpretation of the predictive value of TG for DCE should be conservative given the borderline significance of the relationship in the univariable analysis (*p* = 0.089 in univariable analysis) and the lack of significance in the multivariable analysis.

DHEA-S is the most abundant steroid hormone in the circulatory system. It is almost entirely secreted by the reticular area of the adrenal cortex under the stimulus of adrenocorticotropic hormone (ACTH). Myocardial infarction induces acute physical stress that enhances ACTH and cortisol secretion in the first few hours [35]. As already mentioned, we consider DHEA-S as a marker of acute stress. It was measured within 24 h of the symptom onset in the majority of the patients (78% of whom were diagnosed with STEMI and underwent PCI within 2 h of admission). On the other hand, endogenous levels of DHEA-S have been found to significantly fall after the first day of AMI [35,36]. DHEA-S was found to be lower in severely ill non-coronary patients compared to healthy volunteers in one report [36]. Low cortisol and dehydroepiandrosterone sulfate levels in intensive care unit patients upon admission correlate with the severity of their condition and with risk of death [37]. The decrease in DHEA-S after the first day of AMI does not always correspond to a statistically significant decrease in ACTH and cortisol concentrations [35]. The decrease in DHEA-S in the first days of AMI may be due to the regulation of DHEA-S secretion by factors other than stress hormones [35]. In our study, serum TG was positively associated with high DHEA-S, a factor of survival during critical condition. Correspondingly, very aggressive TG and cholesterol lowering may not be beneficial given that the synthesis of stress hormones and DHEA-S in the adrenal gland is dependent on cholesterol.

Another paradox, complicating the interpretation of our results, is the higher plasma levels of HDL indicated a tendency toward risk of distal coronary embolism in ACS. After adjusting for covariates, this relationship was insignificant. Currently, evidence has emerged that at day 5 of AMI there was a substantial decrease in HDL particle number, and an increase in the number of oxidized HDL [38]. Also, a transient loss of the anti-inflammatory effects of HDL on vascular endothelium has been reported [38]. HDL was measured too early in the course of ACS in our study. Accordingly, their plasma levels are not expected to be influenced by the systemic inflammatory response after PCI. Obviously, incident plasma concentrations of HDL are not independent markers of lower risk of distal coronary embolism at the time of ACS.

We performed an analysis of the association of distal coronary embolism with the distribution of DM in ACS. DM was defined as previously diagnosed and new-onset DM. The results obtained remained non-statistically significant. The results may have been non-significant due to the small total patient group, the small number of events and the correspondingly small number of patients with diabetes. However, cases of hyperglycemia and impaired glucose tolerance in the acute phase of MI were not included in this analysis, although many of these patients have developed overt DM within a year. Long-standing hyperglycemia may augment thrombus formation. Blood glucose, even in the normal range, was found to be an independent predictor of platelet-dependent thrombosis [39]. In prior studies, a high triglyceride–glucose index was associated with increased thrombus burden and with residual coronary ischemia after PCI [40,41]. In addition to the enhanced platelet aggregation and platelet-dependent steps of thrombosis, hyperglycemia inhibits endothelium-dependent vasodilation and decreases nitric oxide levels [9,10]. Acute and persistent hyperglycemia increases intercellular adhesion molecule-1 levels and other inflammatory molecules (tumor necrosis factor-α, CRP), which would augment plugging of platelets and leukocytes in the capillaries [42,43,44].

Several factors have also been proved to be associated with impaired reperfusion during PCI in patients with renal dysfunction. Chronic kidney disease (CKD) can cause platelet activation (significantly higher platelet volume) and increased coagulation [9,10]. Plaques with a higher lipid index are much more common in CKD than in non-CKD patients [9,10]. They have the potential to microrupture and become a source of distal coronary embolization. Studies have shown that coronary reserve (coronary dilation capacity) is reduced in patients with non-obstructive coronary disease and renal dysfunction. Leukocytosis and neutrophil/lymphocyte ratio are higher in patients with stage 3 chronic kidney disease than in those with stage 1 or 2 CKD [9,10]. In our analysis, renal function as assessed using the glomerular filtration rate was not associated with slow blood flow in the IRA after PCI, probably in light of the small differences in the renal function of our study population.

### Limitations

Given the observational design of this study and the small number of distal coronary embolism cases (*n* = 13), its limitations are related to potential selection bias and limited statistical power. Additionally, drawing causal inferences from observational associations challenges further direct application of the conclusions to similar populations of patients.

## 5. Conclusions

Patients with ACS with very high triglyceride levels may be at risk of developing slow blood flow after percutaneous intervention with stent implantation at the time of ACS. Elevated DHEA-S possibly reflects sympathoadrenal, hypothalamus/pituitary/adrenal activity associated with acute coronary syndrome and coronary intervention and thus could have prognostic value.

## Figures and Tables

**Table 1:** Myocardial blush grading.

Myocardial Blush	Angiographic Contrast	
Grades	Angiographic contrast progression	Washout phase
MBG 0	No myocardial blush	No myocardial blush
MBG 1	Slow entry, failure to exit the microvasculature	Dye staining—present on the next injection
MBG 2	Delayed entry into the microvasculature	Dye staining—only minimally diminished in intensity
MBG 3	Ground-glass appearance (“blush”) or opacification of the myocardium in the distribution of the culprit lesion	Opacification is cleared normally. Only mildly/moderately persistent [18]

**Table 2:** Clinical and laboratory characteristics of the patients.

Variable	Mean ± SD
Age, years	64.5 ± 12.3
Sex (men/women)	87 (64%)/50 (36%)
Hypertension	134 (96.4%)
Diabetes mellitus	43 (30.9%)
BMI, kg/m^2^	28.5 ± 5.0
Weight, kg	83.9 ± 16.1
Distal coronary embolism, *n* (%)	13 (9.4%)
STEMI, *n* (%)	109 (78.4%)
NSTEMI, *n* (%)	16 (11.5%)
Unstable angina, *n* (%)	14 (10.1%)
1-vessel CAD, *n* (%)	45 (32.4%)
2-vessel CAD, *n* (%)	52 (37.4%)
3-vessel CAD, *n* (%)	42 (30.2%)
Syntax score	14.9 ± 8.2
Gensini score	46.7±38.9
Prior AMI, *n* (%)	15 (10.8%)
Atrial fibrillation, *n* (%)	18 (12.9%)
Statin, *n* (%)	13 (10.4%)
E2, pmol/L	134.2 ± 93.3
E2/T	0.6 ± 1.2
DHEA-S, µmol/L	3.2 ± 2.3
GFR, ml/min/m^2^	73.8 ± 66.2
PLT × 10^9^/L	248.2 ± 68.4
Glucose, mmol/L	10.9 ± 13.8
WBC × 10^9^/L	10.2 ± 3.5
CRP, mg/L	33.9 ± 77.7
Total cholesterol, mmol/L	5.2 ± 1.3
HDL, mmol/L	1.3 ± 0.5
LDL, mmol/L	3.1 ± 1.2
TG, mmol/L	1.6 ± 0.7
oxLDL, ng/mL	9.4 ± 6.5
Left ventricular EF, %	53.3 ± 10.9
CK, U/L	865.9 ± 1238.5
CK-MB, U/L	93.5 ± 135.8
hsTnT, ng/mL	2.1 ± 2.9

BMI, body mass index; STEMI, ST elevation myocardial infarction; NSTEMI, non-ST elevation myocardial infarction; CAD, coronary artery disease; E2, endogenous 17β-estradiol; T, total testosterone; E2/T, estradiol-to-testosterone ratio; DHEA-S, dehydroepiandrosterone sulfate; PLT, platelet count; WBC, white blood cell count; CRP, C-reactive protein; EF, ejection fraction; HDL, high-density lipoprotein; LDL, low-density lipoprotein; TG, triglycerides; oxLDL, oxidized low-density lipoprotein; CK, creatine kinase; CK-MB, muscle–brain fraction of CK; hsTnT, high-sensitivity troponin T.

**Table 3:** Factors of distal coronary embolism and slow coronary flow at PCI in acute coronary syndrome.

Variable	Uncomplicated PCI	Distal Coronary Embolism	*p*	OR	95% CI	*p*
Age, years	63.3 ± 12.2	63.6 ± 10.1	0.844	0.993	0.948–1.041	0.782
Male patients	80 (64%)	9 (64.3%)	NS			
Diabetes mellitus	13 (2.6%)	6 (1.2%)	NS			
Statin	12 (10.7%)	1 (7.8%)	NS			
E2	131.9 ± 96.8	155.2 ± 59.7	0.077 **	1.002	0.997–1.008	0.436
E2/T	0.6 ± 1.3	0.2 ± 0.2	0.006 *	0.303	0.045–2.055	0.222
DHEA-S	2.9 ± 2.2	4.8 ± 2.6	0.013 *	1.316	1.044–1.659	0.020 *
BMI	28.4 ± 51.1	28.1 ± 3.2	0.825	0.980	0.862–1.116	0.750
GFR, ml/min/m^2^	74.7 ± 69.4	65.2 ± 22.5	0.419	0.993	0.970–1.018	0.595
PLT	250.8 ± 69.3	225.7 ± 57.7	0.212	0.990	0.980–1.000	0.210
WBC	10.1 ± 3.3	11.8 ± 4.8	0.095 **	1.127	0.976–1.016	0.775
CRP	31.9 ± 79.7	51.8 ± 56.5	0.042 *	1.002	0.997–1.007	0.412
Glucose	8.1 ± 3.5	10.0 ± 3.5	0.064 **	1.130	0.990–1.300	0.072 **
TG-glucose index	4.9 ± 0.3	5.0 ± 0.4	0.445	1.940	0.360–10.480	0.443
Total cholesterol	5.3 ± 1.3	5.1 ± 1.6	0.706	0.920	0.572–1.480	0.730
HDL	1.3 ± 0.4	1.6 ± 1.0	0.037 *	2.326	0.918–5.897	0.075 **
LDL	3.1 ± 1.2	2.9 ± 1.2	0.667	0.896	0.544–1.475	0.665
TG	1.5 ± 0.7	1.9 ± 1.1	0.095 **	1.809	0.913–3.583	0.089 **
TG/HDL	1.3 ± 0.7	1.6 ± 1.2	0.820	1.390	0.760–2.540	0.284
oxLDL	9.6 ± 6.6	7.8 ± 6.2	0.447	0.954	0.822–1.107	0.533
SYNTAX score	15.0 ± 8.4	14.5 ± 5.8	0.835	0.992	0.925–1.065	0.834
Gensini score	46.4 ± 39.1	49.6 ± 39.4	0.776	1.002	0.989–1.016	0.778
LV EF%	53.3 ± 10.7	50.7 ± 9.5	0.215	0.975	0.925–1.029	0.357

The abbreviations are the same as those used in Table 2. * denotes significant associations. ** denotes a tendency toward significant associations.

**Table 4:** Variables indicating higher levels of DHEA-S.

	DHEA-S					
Variable	Quartile 1	Quartile 4	*p*	OR	95% CI	*p*
Age	71.3 ± 9.5	56.2 ± 10.9	0.0001 *	0.880	0.827–0.955	0.001 *
E2	112.2 ± 141.6	168.8 ± 79.9	<0.0001 *	1.010	1.000–1.010	0.109
E2/T	0.9 ± 1.6	0.2 ± 0.3	0.016 *	0.140	0.018–0.713	0.020 *
BMI	28.5 ± 5.4	30.2 ± 4.4	0.236	1.087	0.944–1.252	0.232
Weight	84.4 ± 17.5	89.3 ± 16.3	0.468	1.031	0.993–1.071	0.109
GFR	57.4 ± 17.4	79.9 ± 30.5	0.003 *	1.056	1.013–1.102	0.002 *
WBC	9.3 ± 3.1	10.3 ± 3.0	0.254	1.062	0.873–1.291	0.549
CRP	18.3 ± 24.4	35.7 ± 43.8	0.022 *	1.022	0.994–1.049	0.064 **
Total cholesterol	5.1 ± 1.4	5.3 ± 1.3	0.548	1.140	0.750–1.750	0.542
HDL	1.4 ± 0.4	1.5 ± 0.8	0.606	1.290	0.500–3.340	0.604
LDL	3.0 ± 1.3	3.2 ± 1.2	0.739	1.080	0.690–1.610	0.734
TG	1.5 ± 0.6	1.9 ± 0.9	0.034 *	2.320	1.018–5.270	0.045 *
TG/HDL	1.3 ± 1.0	1.6 ± 0.9	0.187	1.350	0.730–2.500	0.334
oxLDL	1.3 ± 1.0	1.3 ± 1.0	0.265	0.939	0.855–1.031	0.186
Gluc	8.3 ± 3.7	9.3 ± 3.7	0.386	1.070	0.920–1.250	0.380
TG-Glucose index	4.9 ± 0.3	5 ± 0.3	0.082 **	5.580	0.780–39.99	0.087 **
Syntax score	14.5 ± 7.8	15.5 ± 6.6	0.618	1.001	0.922–1.086	0.989
Gensini score	58.1 ± 63.1	53.3 ± 34.4	0.734	0.996	0.984–1.008	0.489
LV EF	54.1 ± 12.6	52.7 ± 9.9	0.654	0.979	0.927–1.033	0.431
CK	378.6 ± 615.2	1440.2 ± 1379.5	0.001 *	1.001	1.000–1.002	0.011 *
CK-MB	39.7 ± 56.9	169.8 ± 161.2	0.001 *	1.013	1.003–1.022	0.010 *
hsTnT	0.4 ± 0.7	4.3 ± 3.8	<0.0001 *	3.048	1.423–6.527	0.004 *

The abbreviations are the same as those used in Table 1. * denotes significant associations. ** denotes a tendency toward significant associations.

## Data Availability

Data are available from the corresponding author; details supporting the reported results can be sent to the editors and reviewers upon request.

## Abbreviations

The following abbreviations are used in this manuscript:

ACS	Acute coronary syndrome
AMI	Acute myocardial infarction
STEMI	Acute myocardial infarction with persisting ST elevation
NSTEMI	Acute myocardial infarction with non-persisting ST elevation
CAD	Coronary artery disease
CAG	Coronary angiography
IRA	Infarct-related artery
PCI	Percutaneous coronary intervention
TIMI	Thrombolysis in myocardial infarction
MBG	Myocardial blush grade
GFR	Glomerular filtration rate
CK	Creatine kinase
CPK-MB	Muscle–brain fraction of CK
hs TnT	High-sensitivity troponin T
E2	Total 17β-estradiol
T	Total testosterone
DHEA-S	Dehydroepiandrosterone sulfate
ACTH	Adrenocorticotropic hormone
CRP	C-reactive protein
oxLDL	Oxidized low-density lipoproteins
HDL	High-density lipoprotein
LDL	Low-density lipoprotein
TG	Triglyceride
oxLDL	Oxidized low-density lipoprotein
PLT	Platelets
WBC	White blood cell count
EF	Ejection fraction
GNT	Glyceryl trinitrate

## Appendix A

**Table A1:** Cases of distal coronary artery embolism, final coronary flow and EF, and intracoronary medications used.

Patient	Age, Sex	CAD Type	IRA	TIMI Flow Grade	MBG	Intracoronary Vasodilator	Stent Type	EF, %
1	63 y, man	1-vessel	LADprox	3	2	Verapamil; GTN 400 µg	BMS	42
2	70 y, man	3-vessel	RCAprox	3	-	Eptifibatide 14 mg + 6 mg GTN 400 µg; nitroprusside	BMS	49
3	78 y, man	2-vessel	LADmid	3	-	Verapamil; Eptifibatide 14 mg + 6 mg; GTN Nitroprusside	BMS	64
4	48 y, man	2-vessel	LADprox	3		Verapamil; GTN	BMS	50
5	59 y, man	2-vessel	RCAprox	3	-	GTN 100 µg	BMS	48
6	63 y, man	3-vessel	LAD	2-3	-	GTN 100 µg	BMS	54
7	64 y, man	1-vessel	LAD	2-3	-	Verapamil 200 µg	BMS	48
8	57 y, man	1-vessel	LADmid	3	-	Verapamil 100 µg; GTN 300 µg	DES	59
9	72 y, woman	1-vessel	RCAprox	3	-	None	BMS	48
10	65 y, woman	1-vessel	LADprox	3	-	Verapamil; GTN; Nitroprusside	BMS	48
11	75 y, woman	3-vessel	RCA	3	2–3	GTN 100 µg	BMS	51
12	75 y, woman	1-vessel	LADprox	3	-	GTN 200 µg; Eptifibatide 4 mg	DES	66
13	85 y, woman	1-vessel	RCAprox	3	-	Nitroprusside	BMS	58

Abbreviations: CAD, coronary artery disease; IRA, infarct-related coronary artery; RCA, right coronary artery; LAD, left anterior descending coronary artery; prox, proximal segment; mid, middle segment; TIMI flow grade, Thrombolysis in Myocardial Infarction flow grade; MBG, myocardial blush grade; BMS, bare metal stent; DES, drug-eluding stent.
